
JOURNAL INFORMATION
==============================
NLM Title Abbreviation: Wirtschaftsdienst
Journal ID: Wirtschaftsdienst
NLM Journal ID: 101086612

Wirtschaftsdienst (Hamburg, Germany : 1949)

ISSN: 0043-6275

ARTICLE INFORMATION
==============================
PMCID: PMC7237607
PMID: 32454540
DOI: 10.1007/s10273-020-2647-x
Article ID: 2647
Article version: 1
Subjects: Zeitgespräch

Multilaterale Abkommen: Enthusiasmus und Enttäuschung

Kolev Galina kolev@iwkoeln.de

Matthes Jürgen matthes@iwkoeln.de

grid.473597.b 0000 0000 9854 4639 Institut der deutschen Wirtschaft Köln e.V., Konrad-Adenauer-Ufer 21, 50668 Köln, Deutschland
Electronic publication date: 2020 May 20
Print publication date: 2020
Volume: 100
Issue: 5
First page: 320
Last page: 324
Copyright: © Der/die Autor(en) 2020
License: Open Access Dieser Artikel wird unter der Creative Commons Namensnennung 4.0 International Lizenz veröffentlicht, welche die Nutzung, Vervielfältigung, Bearbeitung, Verbreitung und Wiedergabe in jeglichem Medium und Format erlaubt, sofern Sie den/die ursprünglichen Autor(en) und die Quelle ordnungsgemäß nennen, einen Link zur Creative Commons Lizenz beifügen und angeben, ob Änderungen vorgenommen wurden.
Die in diesem Artikel enthaltenen Bilder und sonstiges Drittmaterial unterliegen ebenfalls der genannten Creative Commons Lizenz, sofern sich aus der Abbildungslegende nichts anderes ergibt. Sofern das betreffende Material nicht unter der genannten Creative Commons Lizenz steht und die betreffende Handlung nicht nach gesetzlichen Vorschriften erlaubt ist, ist für die oben aufgeführten Weiterverwendungen des Materials die Einwilligung des jeweiligen Rechteinhabers einzuholen.
Weitere Details zur Lizenz entnehmen Sie bitte der Lizenzinformation auf http://creativecommons.org/licenses/by/4.0/deed.de.
License URL: https://creativecommons.org/licenses/by/4.0/

issue-copyright-statement: © The Author(s) 2020

==============================
Prof. Dr. Galina Kolev leitet die Forschungsgruppe Gesamtwirtschaftliche Analysen und Konjunktur am Institut der deutschen Wirtschaft (IW) in Köln und ist Professorin für VWL an der Hochschule RheinMain in Wiesbaden.

Die multilaterale Welthandelsordnung steht unter erheblichem Stress. Obwohl sie in längerfristiger Perspektive ein Erfolgsmodell ist, wurde die Welthandelsorganisation (WTO) in den letzten Jahren in mehrfacher Hinsicht geschwächt. Das Scheitern der Doha-Welthandelsrunde nach letzten Wiederbelebungsversuchen im Jahr 2008 hat gezeigt, dass sie ihrer Funktion, die Liberalisierung der Handelsregeln voranzubringen, nicht mehr hinreichend gerecht wird. Zudem ist ihre Funktion zur Durchsetzung des bestehenden Regelwerks empfindlich geschwächt, seit die WTO-Berufungsinstanz außer Kraft gesetzt wurde. Darüber hinaus haben protektionistische Tendenzen deutlich zugenommen. Seit Ende 2008 wurden etwa 18.000 handelshemmende Maßnahmen weltweit registriert und somit mehr als doppelt so viele wie die Zahl der handelsliberalisierenden Maßnahmen (Global Trade Alert, 2020). Auch die Globalisierungskritik wirkt bremsend auf die Weiterentwicklung der Handelsregeln und begünstigt den neuen Protektionismus.

In der aktuellen Corona-Pandemie zeigt sich weitere Skepsis bezüglich der Rolle der WTO als internationale Struktur zur Lösung von globalen Problemen. James Bacchus (2020) betont, dass die G20-Länder zwar auf die internationale Zusammenarbeit setzen, um die Folgen der Virusausbreitung einzudämmen. Einzig die WTO wird jedoch in der gemeinsamen Erklärung der G20 nicht erwähnt.

Unter Handelsexperten gibt es jedoch bereits konkrete Vorschläge, wie die WTO einen wichtigen Beitrag zur Bekämpfung der Pandemie leisten kann. Ganz oben auf der Liste sieht Bacchus (2020) die Notwendigkeit, Exportverbote für Medikamente und medizinische Versorgung zurückzunehmen und zu unterlassen. Mittlerweile hat die WTO solche Maßnahmen in 80 Ländern weltweit festgestellt (WTO, 2020a). Diese Maßnahmen können je nach Anwendung nach den WTO-Regeln legal sein, stehen jedoch im Widerspruch zum Ziel der G20, die Maßnahmen zu koordinieren, sodass unnötige Eingriffe in den internationalen Verkehr und Handel vermieden werden (Bacchus, 2020). Denn diese Eingriffe verhindern, dass begrenzte Medikamente und Vorräte dorthin gelangen, wo sie für einen wirksamen koordinierten globalen Kampf gegen das globale Coronavirus am dringendsten benötigt werden.

### Zölle für medizinische Produkte

Eine weitere Baustelle für die Handelspolitik stellen die Zölle, die viele Länder auf eine ganze Reihe von medizinischen Produkten erheben, die während der Pandemie überall benötigt werden, dar (Bacchus, 2020). Alle Länder müssen mindestens einen Teil dieser Waren importieren und kaum ein Land kann die gesamte Menge an Waren in den Mengen produzieren, die es möglicherweise benötigt. Zölle auf lebensrettende Güter können somit nicht sinnvoll sein, da sie künstlich einen Engpass für diese Güter erzeugen.

Bacchus (2020) schlägt weitere Maßnahmen im Zusammenhang mit der Virusbekämpfung vor, die auf WTO-Ebene initiiert werden können:

- Förderung der Transparenz bei allen nationalen Maßnahmen zur Bekämpfung des Virus;
- Verzicht auf „Buy Local“-Anforderungen, die die Preise für staatliche Einkäufe in weiten Teilen der Welt erhöhen;
- Verbesserung der Handelserleichterungen zur Verringerung der Bürokratiekosten für den Handel mit gesundheitsbezogenen Produkten;
- Beseitigung nichttarifärer Handelshemmnisse, die den Handel mit Arzneimitteln und medizinischen Geräten behindern;
- Annahme internationaler Standards zur Gewährleistung der Sicherheit und Qualität importierter Medizinprodukte;
- Genehmigung von Subventionen für die Herstellung der neuen Medikamente, die dringend benötigt werden, um das Coronavirus zu stoppen;
- Bekräftigung, dass die WTO-Regeln die Zwangslizenzierung der benötigten Arzneimittel durch Entwicklungsländer unter diesen schlimmen Umständen ermöglichen;
- Erleichterung für Beschäftigte im Gesundheitswesen, sich über Grenzen hinweg zu bewegen.

Einige der vorgeschlagenen Maßnahmen finden bereits in der handelspolitischen Praxis Anwendung. So veröffentlichte die Europäische Kommission (2020) Anfang April 2020 den Beschluss, die Einfuhr von medizinischer Ausrüstung aus Nicht-EU-Ländern von Zöllen und Mehrwertsteuer gemäß Verordnung (EG) Nr. 1186/2009 des Rats und Richtlinie 2009/132/EG des Rats zu befreien, um die Belieferung von Ärzten, Pflegepersonal und Patienten mit dringend benötigter medizinischer Ausrüstung finanziell zu erleichtern. Die Entscheidung betrifft Gegenstände wie Schutzmasken, Testkits, Beatmungsgeräte und andere medizinische Ausrüstung und ist vorerst für einen Zeitraum von sechs Monaten gültig.

### Rolle der WTO in der Corona-Krise

Im Zusammenhang mit der aktuellen Krise hat die WTO (2020b) im Rahmen eines Berichts die Rolle von handelspolitischen Maßnahmen im Bereich der Medizinprodukte beleuchtet und ihre eigene Rolle im Prozess der Handelsliberalisierung betont. Laut diesem Bericht entfielen im Jahr 2019 etwa 5 % des globalen Warenhandels oder etwa 2.000 Mrd. US-$ auf den Handel mit Medizinprodukten; 1,7 % des globalen Handels oder knapp 600 Mrd. US-$ umfassen den Handel mit als „kritisch“ zu bezeichnenden Produkten, bei denen in der Corona-Krise erhebliche Engpässe verzeichnet wurden. Die WTO hat auf drei Wegen dazu beigetragen, dass der Handel mit Medizin- und Pharmaprodukten liberalisiert wird:

1. durch die allgemeinen Beschlüsse bei der Gründung der WTO im Jahr 1995;
2. durch den Abschluss des plurilateralen sektoralen Abkommens über pharmazeutische Produkte (das sogenannte „zero for zero“-Abkommen oder auch „Pharma-Abkommen“ genannt, zu dem aktuell Kanada, die EU, Japan, Macao/China, Norwegen, die Schweiz und die USA gehören) in der Uruguay-Runde und seinen vier nachfolgenden Revisionen;
3. sowie durch die Erweiterung des Informationstechnologie-Abkommens im Jahr 2015 (die sogenannte ITA-exp, die für etwa 80 % der technologieintensiven medizinischen Ausrüstung die Zölle abschaffen und die Importkosten bis 2023 senken soll – jedoch nur unter den Teilnehmern des Abkommens, also unter Ländern, die sowieso bereits einen durchschnittlichen Zollsatz in Höhe von 0,4 % erheben).

Im Jahr 2019 war der durchschnittliche angewandte Zollsatz auf Medizinprodukte mit 4,8 % wesentlich niedriger als der durchschnittliche Zollsatz auf Nichtagrargüter (WTO, 2020b). In einigen Ländern, wie z. B. Island, Hongkong oder Singapur, werden Medizinprodukte sogar zollfrei importiert. In der EU beträgt der durchschnittliche Zollsatz gegenüber Drittländern 1,5 %. Nichtsdestotrotz bleiben die Zollsätze auf viele Produkte relativ hoch:

- Der durchschnittliche angewandte Zollsatz für Seife beträgt 17 %, bei einzelnen WTO-Mitgliedstaaten liegt er sogar bei 65 %. Mehr als die Hälfte der WTO-Mitgliedstaaten wenden einen Zollsatz von über 50 % an.

- Schutzausrüstungen werden mit einen durchschnittlichen Zollsatz in Höhe von 11,5 % belegt, in manchen Ländern werden die Importe im Durchschnitt mit 27 % verzollt. Über 40 % der Länder belegen die Importe von Schutzmasken mit einem Zollsatz von über 10 %.

- Relativ hoch mit 6,2 % sind die Zollsätze auf Krankenhaus- und Labormaterialien.

Bemerkenswert ist weiterhin, dass es bei Medizinprodukten eine relativ große Lücke (water in the tariff) zwischen dem Zollsatz gibt, zu dem sich die Länder in der WTO als Maximalzollsatz verpflichtet haben (bound) und dem tatsächlich angewandten Zollsatz (WTO, 2020b). Die WTO sieht das als Potenzial, die offiziell vereinbarten Grenzen für die Zollsätze zu senken. Es ist jedoch auch denkbar, dass einzelne Länder diese Lücke nutzen, um nach der Corona-Krise über die handelspolitische Schiene den Aufbau von inländischen Produktionskapazitäten voranzutreiben, ohne gegen WTO-Vereinbarungen zu verstoßen.

Des Weiteren weist die WTO (2020b) darauf hin, dass 35 % des weltweiten Angebots von Medizinprodukten aus Deutschland (14 %), den USA (12 %) und der Schweiz (9 %) stammen. Die USA und Deutschland sind mit 19 % und 9 % die größten Importeure von Medizinprodukten, gefolgt von China mit 6 %. Rechnet man die EU-Länder zusammen, so rangiert die EU sowohl was die Exporte als auch was die Importe angeht auf dem ersten Platz im internationalen Vergleich. Im Bereich der COVID-19-relevanten Produkte liegt diese Lücke bei über 20 %, für die Medizinprodukte insgesamt sind es etwa 17 %.

Die aktuelle Krisensituation offenbart die enorme Relevanz multinationaler Organisationen wie der WTO, um die internationale Kooperation zu stärken und globale Lösungen für globale Probleme zu suchen. Aus heutiger Sicht ist es jedoch alles andere als klar, ob in Zukunft die Länder weltweit ihre Zusammenarbeit in handelspolitischen Fragen ausbauen und den Multilateralismus wieder beleben werden, zumal die Unterbrechung der internationalen Wertschöpfungsketten als Folge der Virusausbreitung eine Neubewertung der mit der internationalen Arbeitsteilung verbundenen Risiken und Chancen notwendig gemacht hat. Es ist nicht ausgeschlossen, dass nach der vorübergehenden Reduktion der Handelshemmnisse im Handel mit Medizinprodukten eine neue Welle von protektionistischen Maßnahmen folgt, um wichtige Branchen vor ausländischem Wettbewerb zu schützen und Produktionskapazitäten im Inland aufzubauen. Hierbei ist es von entscheidender Bedeutung, solche Eingriffe auf das Minimum zu beschränken und in jedem Fall genau abzuwägen, ob es sich in der Tat um einen Bereich handelt, der zu der Versorgung der Bevölkerung mit lebensnotwendigen Gütern gehört. Das Gebot der Stunde soll es jedoch sein, die bereits gestörten Lieferketten nicht durch zusätzliche Handelskonflikte weiter zu beeinträchtigen (Kolev und Obst, 2020).

## Ursachen für die Probleme der WTO

Jenseits des Blicks auf die aktuelle Situation stellt sich die Frage, was die tieferen Ursachen für die eingangs aufgezeigten Probleme der WTO sind und wie eine erfolgreiche Zukunft der multilateralen Handelsordnung gesichert werden kann. In der Phase um und nach der Gründung der WTO im Jahr 1995 herrschte allenthalben Enthusiasmus mit Blick auf die internationale Handelspolitik. Die Uruguay-Runde war erfolgreich abgeschlossen und setzte einen Liberalisierungsschub auf breiter Front in Gang (Zollsenkungen, Integration von Agrar- und Textilhandel sowie Dienstleistungen und geistige Eigentumsrechte in die Handelsordnung). Die Marktwirtschaft schien gegen die Planwirtschaft gesiegt zu haben und die Globalisierung war immer weiter auf dem Vormarsch. Mitte der 1990er Jahre wurde in Europa der Binnenmarkt weiterentwickelt, die Euro-Einführung vorbereitet und auch die handelspolitischen Weichen für die Osterweiterung gestellt. Das Credo von Liberalisierung und Offenheit schien unangefochten zu dominieren.

Doch schon um die Jahrtausendwende zeigten sich erste Sollbruchstellen, die im weiteren Zeitverlauf zunehmend aufbrechen sollten. Zivilgesellschaftliche Globalisierungskritik nahm immer weiter zu, wie das Scheitern der WTO-Ministerkonferenz in Seattle bereits 1999 zeigte. Zwar wurde 2001 die Doha-Runde begonnen und China in die WTO aufgenommen. Doch der Konkurrenzdruck, der in der Folge durch sehr schnell steigende chinesische Exporte weltweit entstand, wurde schon bald zu einer der Ursachen für das spätere Scheitern der Doha-Runde (Matthes, 2006).

Darüber hinaus erwies sich paradoxerweise ein wichtiger Erfolg der Uruguay-Runde als zentraler Hemmschuh für die weitere Handelsliberalisierung. Denn mit der Gründung der WTO wurde das Konsensprinzip eingeführt. Damit erhielt jedes einzelne Entwicklungs- und Schwellenland als Gegenleistung für die stärkere Bindungskraft der Handelsregeln eine Vetomöglichkeit. Gerade die großen aufstrebenden Schwellenländer wie China, Indien und Brasilien münzten zudem ihre wirtschaftlichen Erfolge in zunehmendes Selbstbewusstsein in der WTO um. Sie verweigerten die weitere Liberalisierung des Industriegüterhandels, weil die Industrieländer aus ihrer Sicht ihre Agrar- und Textilmärkte nicht weit genug liberalisieren wollten. Vor allem an dieser Konfrontation scheiterte die Doha-Runde.

Die Unternehmen in den Industrieländern drängten trotz der stockenden Doha-Runde auf eine Erweiterung der Handelsregeln, um ihre Globalisierungsstrategien besser abzusichern. In Reaktion auf diesen politischen Druck wichen immer mehr Industrieländer vom erstbesten multilateralen Weg ab und setzten auf zumeist bilaterale und vereinzelt auch regionale Handelsabkommen. Die Zahl bilateraler Handelsabkommen nahm in der Folge vor allem nach der Jahrtausendwende rasant zu, als auch die USA auf diesen Kurs einschwenkten. Auch in den Entwicklungs- und Schwellenländern setzte sich dieser Trend zunehmend durch. Dies verringerte jedoch den Lobbydruck der exportorientierten Wirtschaft bei der WTO in Genf und trug auf diese Weise mit zum Scheitern der Doha-Runde bei, da die Politik weniger Druck verspürte, die Widerstände der Agrarlobbies in den Industrieländern zu brechen. Zudem schafft das komplexe Netzwerk bilateraler Freihandelsabkommen (Spaghetti Bowl) eine vor allem für kleinere Unternehmen schwer handhabbare Bürokratie. Es wäre daher wünschenswert, wenn es gelänge, ähnliche bilaterale Abkommen zwischen mehreren Staaten zu plurilateralen Abkommen zusammenzufassen und dabei die Regeln möglichst zu vereinheitlichen.

Wie eingangs erwähnt ist die Außerkraftsetzung der Berufungsinstanz ihres Streitschlichtungsmechanismus ein schwerer Schlag für die WTO. Die USA haben so lange die Ernennung neuer Mitglieder des Appellate Body blockiert, bis dieser aufgrund einer nicht mehr ausreichenden Besetzung handlungsunfähig wurde. Dieses Vorgehen ging einher mit einer zunehmend protektionistischen Ausrichtung der US-Handelspolitik, vor allem gegenüber China, aber auch gegenüber anderen Handelspartnern wie der EU. Besonders kritisch zu sehen ist dabei, dass sich die US-Administration auf eine vermeintliche Gefährdung der nationalen Sicherheit beruft, um die Erhebung von Strafzöllen auf Stahl- und Aluminiumimporte von NATO-Verbündeten und potenzielle Handelsbarrieren gegen Automobilimporte zu rechtfertigen. Die WTO-Regeln (Artikel XXI) sehen recht weitgehende Schutzmöglichkeiten für die Sicherstellung der nationalen Sicherheit vor. Dieses potenzielle Schlupfloch für protektionistische Schutzmöglichkeiten wurde bislang in einer Art Gentlemen‘s Agreement von den WTO-Mitgliedern bewusst kaum genutzt. Die skrupellose Berufung der USA auf diese Klausel könnte daher zu einem Dammbruch führen.

Die USA haben den Appellate Body wohl auch deshalb handlungsunfähig gemacht, damit ihr bedenkliches Vorgehen nicht doch durch die WTO als rechtskräftig verurteilt werden kann. Doch neben weiteren eher technischen Gründen liegt eine wesentliche Ursache der US-Blockadepolitik darin, dass die US-Administration dem Appellate Body vorwerfen, die WTO-Regeln aus ihrer Sicht fragwürdig ausgelegt zu haben. Es wird vor allem kritisiert, dass die Nutzung von Handelsschutzinstrumenten zu sehr eingeschränkt wurde. Bei Antisubventionsmaßnahmen z. B. ist es durch die Auslegung des Appellate Body sehr schwer geworden, wettbewerbsverzerrendes Verhalten von chinesischen Staatsunternehmen einzuhegen (European Parliament, 2019). Nach Ansicht der USA hat der Appellate Body seine Kompetenzen damit überschritten, weil er nicht die institutionelle Stellung eines unabhängigen nationalen Gerichts habe, das über Präzedenzfälle Richterrecht schafft, sondern sich sehr eng an die von den souveränen Mitgliedstaaten gesetzten Regeln halten müsse. Diese Kritik erscheint durchaus nachvollziehbar.

## Die Rolle Chinas in der Welthandelspolitik

Im Zusammenhang mit den angesprochenen Handelspraktiken Chinas ist zu kritisieren, dass die WTO-Regeln zu große Lücken aufweisen, um die zunehmenden Auswirkungen des chinesischen Staatskapitalismus auf die Weltmärkte einzuhegen. Hier liegt wohl der größte Kritikpunkt an der WTO und die Kernursache für die aggressive Handelspolitik der USA gegenüber China und der WTO. Es geht insbesondere um Wettbewerbsverzerrungen durch vielfältige industriepolitische Subventionen, Staatsunternehmen und resultierende Überkapazitäten. Zudem bedient sich China verschiedener Praktiken eines forcierten Technologietransfers, vor allem im Rahmen von erzwungenen Joint Ventures als Vorbedingungen für den Marktzugang in China. Zwar hat China hier zuletzt verschiedene Liberalisierungsmaßnahmen ergriffen, etwa im Rahmen des im Jahr 2020 in Kraft getretenen Investitionsgesetzes. Viele Bestimmungen bleiben dabei jedoch vage und es ist fraglich, ob sie bei nicht unabhängigen Gerichten im Zweifelsfall erfolgreich einklagbar wären. Zudem spricht einiges dafür, dass die Liberalisierungsmaßnahmen auch als Reaktion auf den massiven Druck der USA im Zuge des Handelskriegs gegenüber China umgesetzt wurden.

In der WTO hat sich China jedoch schon seit längerem den Versuchen der EU und der USA widersetzt, die WTO-Regeln zu reformieren, um die chinesischen Handelspraktiken einzuhegen. Dies betrifft selbst die Durchsetzung der WTO-Notifikationsvorgaben für Subventionen. In Reaktion auf die Blockadehaltung Chinas haben die EU, die USA und Japan im Rahmen einer trilateralen Initiative verschiedene Reformvorschläge gemacht. Einen Vorschlag zur Verbesserung der Notifikationsdisziplin haben sich weitere Staaten angeschlossen und diesen gemeinsam in die WTO eingebracht. Anfang 2020 hat die trilaterale Initiative zudem umfangreiche Regelverschärfungen für industrielle Subventionen vorgeschlagen. Es ist in diesem Zusammenhang im Übrigen bemerkenswert, dass sich die USA auf dieser Ebene durchaus konstruktiv in der WTO engagieren. Ohne die Kooperation Chinas wird es aufgrund des Konsensprinzips in der WTO jedoch nicht zu Reformen kommen. Es ist daher richtig, mit der Trilateralen Initiative gemeinsam und auch auf unilateralem Weg auf verschiedene Weise den Verhandlungsdruck auf China zu erhöhen. In diesem Sinne ist auch die Neuausrichtung der EU-Strategie gegenüber China zu begrüßen (Europäische Kommission, 2019).

Als Fazit bleibt festzuhalten: Wenn die Spillovers von Chinas Staatskapitalismus auf den Weltmarkt weiter zunehmen und China weiterhin ausreichende Reformen des WTO-Regelwerks blockiert, wird die WTO aus der Phase der Enttäuschung wohl nicht mehr herauskommen und auf Dauer nur noch in der Erinnerung auf die Zeiten des früheren Enthusiasmus zurückblicken können.


Jürgen Matthes, Dipl.-Volkswirt, leitet das Kompetenzfeld Internationale Wirtschaftsordnung am IW Köln.


Literatur

Bacchus, J. (2020), Governments Should Rely More on the WTO in the Fight Against the Coronavirus, Cato Institute, 3. April, https://www.cato.org/blog/governments-should-rely-more-wto-fight-against-co-ronavirus (8. April 2020).
Europäische Kommission (2019), EU-China — A strategic outlook, Brüssel, 12. März, https://ec.europa.eu/commission/sites/beta-political/files/communication-eu-china-a-strategic-outlook.pdf (17. April 2020).
Europäische Kommission (2020), Coronakrise: Kommission befreit Einfuhr von medizinischer Ausrüstung aus Nicht-EU-Ländern von Zöllen und Mehrwertsteuer, Pressemitteilung, 3. April, https://ec.europa.eu/germany/news/20200403-einfuhr-medizinischer-ausruestung-aus-nicht-eu-laendern_de (8. April 2020).
European Parliament (2019), Balanced and fairer world trade defence — EU, US and WTO perspectives, Workshop documentation, EP/EXPO/B/INTA/2018/08-10, Brüssel.
Global Trade Alert (2020), Global Dynamics, https://www.globaltradealert.org/global_dynamics (11. April 2020).
Kolev, G. und T. Obst (2020), Die Abhängigkeit der deutschen Wirtschaft von internationalen Lieferketten, IW-Report, 16.
Matthes J Doha im Koma ifo-Schnelldienst 2006 59 16 11 14
Welthandelsorganisation (WTO) (2020a), Export Prohibitions and Restrictions, Information Note, https://www.wto.org/english/tratop_e/covid19_e/export_prohibitions_report_e.pdf (24. April 2020).
Welthandelsorganisation (WTO) (2020b), Trade in Medical Goods in the Context of Tackling COVID-19, Information Note, 3. April, https://www.wto.org/english/news_e/news20_e/rese_03apr20_e.pdf (8. April 2020).
